# Chronic inflammatory diseases, subclinical atherosclerosis, and cardiovascular diseases: Design, objectives, and baseline characteristics of a prospective case-cohort study ‒ ELSA-Brasil

**DOI:** 10.1016/j.clinsp.2022.100013

**Published:** 2022-04-06

**Authors:** Isabela M. Bensenor, Alessandra C. Goulart, Alexandre C. Pereira, André R. Brunoni, Airlane Alencar, Raul D. Santos, Márcio S. Bittencourt, Rosa W. Telles, Luciana Andrade Carneiro Machado, Sandhi Maria Barreto, Bianca de Almeida-Pititto, Carolina Porto Silva Janovsky, José Augusto Sgarbi, William R. Tebar, Vandrize Meneghini, Fernando Barbosa Junior, Ana Cristina de Medeiros Ribeiro, Sandra Gofinet Pasoto, Rosa Maria R. Pereira, Eloísa Bonfá, Aytan M. Sipahi, Itamar de S. Santos, Paulo A. Lotufo

**Affiliations:** a Centro de Pesquisa Clínica e Epidemiológica, Hospital Universitário, Universidade de São Paulo (USP), São Paulo, SP, Brazil; b Laboratório de Genética e Cardiologia Molecular, Instituto do Coração de São Paulo (InCor), Hospital das Clínicas HCFMUSP, Faculdade de Medicina, Universidade de São Paulo, São Paulo, SP, Brazil; c Instituto de Matemática e Estatística, Universidade de São Paulo, São Paulo, SP, Brazil; d Unidade Clínica de Lipides, Instituto do Coração (InCor), Hospital das Clínicas HCFMUSP, Faculdade de Medicina, Universidade de São Paulo, São Paulo, SP, Brazil; e Clinics Hospital, UFMG, Belo Horizonte, MG, Brazil; f Faculdade de Medicina, Universidade Federal de Minas Gerais (UFMG), Belo Horizonte, MG, Brazil; g Departamento de Medicina Preventiva, Escola Paulista de Medicina, Universidade Federal de São Paulo, São Paulo, SP, Brazil; h Unidade de Tireoide, Divisão de Endocrinologia e Metabolismo, Faculdade de Medicina de Marília (Famema), Marília, SP, Brazil; i Laboratório de Toxicologia e Essencialidade de Metais, Faculdade de Ciências Farmacêuticas de Ribeirão Preto, USP, Ribeirão Preto, SP, Brazil; j Divisão de Reumatologia, Faculdade de Medicina FMUSP, São Paulo, SP, Brazil; k Laboratório de Metabolismo Ósseo, Reumatologia, Hospital das Clínicas HCFMUSP, Faculdade de Medicina, Universidade de São Paulo, São Paulo, SP, Brazil; l Clínica e Laboratório de Gastroenterologia Experimental (LIM07), Hospital das Clínicas HCFMUSP, Faculdade de Medicina, Universidade de São Paulo, São Paulo, SP, Brazil

**Keywords:** Chronic Inflammatory disease, Cardiovascular disease, Coronary artery calcium (CAC), Carotid intima-media thickness (CIMT), Fatal and non-fatal cardiovascular events

## Abstract

**Objectives:**

This analysis describes the protocol of a study with a case-cohort to design to prospectively evaluate the incidence of subclinical atherosclerosis and Cardiovascular Disease (CVD) in Chronic Inflammatory Disease (CID) participants compared to non-diseased ones.

**Methods:**

A high-risk group for CID was defined based on data collected in all visits on self-reported medical diagnosis, use of medicines, and levels of high-sensitivity C-Reactive Protein >10 mg/L. The comparison group is the Aleatory Cohort Sample (ACS): a group with 10% of participants selected at baseline who represent the entire cohort. In both groups, specific biomarkers for DIC, markers of subclinical atherosclerosis, and CVD morbimortality will be tested using weighted Cox.

**Results:**

The high-risk group (n = 2,949; aged 53.6 ± 9.2; 65.5% women) and the ACS (n=1543; 52.2±8.8; 54.1% women) were identified. Beyond being older and mostly women, participants in the high-risk group present low average income (29.1% vs. 24.8%, p < 0.0001), higher BMI (Kg/m^2^) (28.1 vs. 26.9, p < 0.0001), higher waist circumference (cm) (93.3 vs. 91, p < 0.0001), higher frequencies of hypertension (40.2% vs. 34.5%, p < 0.0001), diabetes (20.7% vs. 17%, p = 0.003) depression (5.8% vs. 3.9%, p = 0.007) and higher levels of GlycA a new inflammatory marker (p < 0.0001) compared to the ACS.

**Conclusions:**

The high-risk group selected mostly women, older, lower-income/education, higher BMI, waist circumference, and of hypertension, diabetes, depression, and higher levels of GlycA when compared to the ACS. The strategy chosen to define the high-risk group seems adequate given that multiple sociodemographic and clinical characteristics are compatible with CID.

## Highlights

• The morbidity and mortality associated with cardiovascular diseases may be higher in patients with chronic inflammatory diseases.
• The lipid paradox has to be studied in all chronic inflammatory diseases.
• The performance of scores of cardiovascular risk may underestimate the real risk in patients with chronic inflammatory diseases.

## Introduction

Cardiovascular Diseases (CVD) are the leading cause of mortality worldwide, with a particular burden in Low-Middle Income Countries (LMIC).^1^^,^^2^ Since the middle of the 20^th^ century, cardiovascular mortality has been the leading cause of death in Brazil,^3^ with a high burden in blacks and people from low socioeconomic status, mainly expressed as a substantial number of premature deaths.^4^

Chronic Inflammatory Diseases (CID) are a group of diseases characterized by a strong inflammatory component, including numerous rheumatic diseases such as rheumatoid arthritis, systemic lupus erythematosus, primary Sjögren's syndrome, primary vasculitis, and also psoriasis. Recently, celiac disease was included as part of the group.^5^

CVD and atherosclerosis have been considered to share many of the hallmarks and physiopathological processes of inflammatory diseases.^6^^,^^7^ Indeed, CVD is a frequent cause of death in patients with CID.^8^, ^9^, ^10^ In addition, several inflammatory steps are an essential part of CVD pathophysiologies, such as endothelial dysfunction, oxidative stress, the accumulation of macrophages and cytokines. Thus, it is not unexpected to hypothesize that the atherosclerotic process, the most critical point in CVD, is probably accelerated in the setting of CID as a comorbidity.^8^, ^9^, ^10^

It is also known that both the use of cardiovascular risk scores such as Framingham or Reynolds is subestimated in patients with rheumatoid arthritis not only because these scores were validated in samples with low cardiovascular risk^11^^,^^12^ but also because of the low levels of LDL-cholesterol in these patients compared to population samples.^8^, ^9^, ^10^ However, these patients presented a higher frequency of cardiovascular events and mortality. This was defined as the lipid paradox: the association of lower risk scores and LDL-cholesterol with a higher frequency of subclinical atherosclerosis,^13^ cardiovascular events, and mortality.^14^

The Brazilian Longitudinal Study of Adult Health (ELSA-Brasil) is an ongoing multicenter prospective cohort study of civil servants followed since 2008‒2010.^15^ The cohort is focused on the study of CVD and diabetes, and associated factors. One of the innovations of the study was the possibility to evaluate not only classical risk factors for CVD but also non-classical risk factors since the former do not explain all CVD events, especially in high-risk groups, as is the case of rheumatoid arthritis and other inflammatory diseases.^10^^,^^16^^,^^17^ Here, the authors describe the study design and protocols as well as baseline characteristics of the sample divided into two groups: the high-risk group for CID and the comparison group of the aleatory cohort sample. The authors of the present study will use this sample to prospectively evaluate the incidence of subclinical atherosclerosis and clinical CVD in the study participants. In addition, the study is an interesting setting to investigate the lipid paradox described in rheumatoid arthritis in other CID.

## Materials and methods

### Design and population study

ELSA-Brasil is a prospective cohort study that follows 15,105 civil servants, 35 to 74 years from six universities and research institutions in different cities in Brazil: Salvador (BA), Vitória (ES), Belo Horizonte (MG), Rio de Janeiro (RJ), São Paulo (SP) and Porto Alegre (RS).^15^^,^^18^, ^19^, ^20^ The ELSA-Brasil protocol was approved in all six centers by the Institutional Review Boards addressing research in human participants, according to the Declaration of Helsinki. Written informed consent was obtained from all participants. According to standard protocols, each participant was interviewed in the workplace, and then they visited the Research Center for clinical interviews and measurements. Interviews and examinations at each site were conducted by trained personnel under strict quality control. After the baseline, the second visit was performed from 2012‒2014, the third visit from 2017‒2019, and a new data collection expected to begin in 2021 was postponed for 2022 or even later depending on the burden of COVID-19 in the country.

### Case-cohort design

For most CID in this analysis, a case-cohort study design will be used. Possible cases identified by prior self-reported medical diagnosis or use of specific medications for the treatment of CID or high-sensitivity C-Reactive Protein (hs-CRP) higher than 10 mg/L in one or more visits were selected to be included in the high-risk group. The comparison group is composed of 1,543 participants of the Aleatory Cohort Sample (ACS) that was selected randomly at the beginning of the baseline examination. It includes 10% of the entire sample, and it is representative of all 15,105 participants in the study. Stored serum samples of both groups of participants will be tested for specific biomarkers for each one of the CID listed as part of the study. The only exception is for psoriasis, which will be confirmed based on a prior medical diagnosis of disease. ELSA-Brasil was strategically designed to permit case-cohort studies. Case-cohort studies are less costly since only a subsample of participants selected independent of the outcomes is included as the comparison group for all ancillary studies that are part of this project. Moreover, also as part of the study strategy, participants selected in the ACS have additional biological samples collected and stored at each visit, which permits the rational use of stored biological samples.^21^

### Sample selection

In the baseline examination (2008‒2010), information was collected about the presence of arthritis without specification, and specifically about rheumatoid arthritis and systemic lupus erythematosus using the question: Have you been previously told by a physician that you had/have arthritis? Rheumatoid arthritis? Systemic lupus erythematosus? The study also included as part of the questionnaire an open question about the previous medical diagnosis of other diseases with the possibility to inform any diseases such as Sjögren's syndrome, vasculitis, scleroderma, Reiter, psoriasis, and several others that were not specifically included in the questionnaire. If a disease was informed, the year of medical diagnosis was also recorded. Information on the celiac disease was captured in the question on reasons for changing the previous diet in the last 6 months at baseline, and from an open question asking about the presence of gastrointestinal symptoms and malabsorption syndromes at baseline.

In the third visit (2017‒2019), the same questions on prior history of rheumatoid arthritis and systemic lupus erythematosus were applied; and a new question about the previous medical diagnosis of psoriasis was introduced, always asking the year of medical diagnosis. The authors also have information on reasons for diet changes in the 6 previous months.

The study also has detailed information about the use of continuous medication in the 15 days before coming to the research center and the use of sporadic medication utilized as pain killers and non-steroidal and steroidal inflammatory medications in the three face-to-face examinations (2008‒2010; 2012‒2014; and 2017‒2019). In addition, the study has information about hs-CRP in all samples in all three visits. Therefore, the high-risk for CID includes participants that self-reported prior medical diagnosis of CID, use of specific medications to treat CID, or high levels of inflammatory biomarkers in at least one of data collection. In addition, all these participants will be tested for specific biomarkers of CID. When the authors obtain the results of all biomarkers, cases of CID will be confirmed based on the presence of self-reported medical diagnosis, use of specific medication to treat the disease, and the presence of biomarkers following the strategy used by Kuller et al. to define rheumatoid arthritis in previous analyses.^8^

### Cohort surveillance and event follow-up

ELSA-Brasil clinical cardiovascular endpoints include acute myocardial infarction, unstable angina pectoris, cardiac revascularization, resuscitated cardiac arrest, heart failure, peripheral arterial disease, atherothrombotic stroke, transient ischemic attack, incident diabetes, and chronic kidney disease. The study also ascertains diabetes-related events (blindness, amputation) and acute complications resulting in hospitalization (ketoacidosis, hyperosmolar state, severe hypoglycemia). In addition, by direct comparison of data from repeated examinations over time, the study also investigates changes in weight, blood pressure, dyslipidemia, and other metabolic disorders, and the occurrence of microalbuminuria, retinopathy, cognitive dysfunction, and psychiatric illnesses by comparison of data from repeated examinations over time.

Surveillance is being conducted through annual telephone interviews, return visits to ELSA‒Brasil clinics (every 4 to 5 years), employer reports, and linkage to national databases, such as the National Mortality System.

Events are classified according to the ELSA-Brasil protocols by a panel of trained physicians from the study. Deaths are identified primarily from reports by next of kin and employers. Underlying and contributing causes of deaths are classified according to death certificates and available hospital records and, for out-of-hospital deaths, according to information obtained from interviews with next of kin and physicians.

### Measurement of biomarkers of inflammatory diseases

In the group of participants with a high risk for CID, and also in the aleatory cohort sample, several biomarkers will be tested: rheumatoid factor (FR LATEX COMPLETO, WAMA, São Carlos, Brazil), anti-cyclic citrullinated peptides antibodies for rheumatoid arthritis (Anti-CCP (IgG- ELISA, Euroimmun, Lübek, Germany); anti-nuclear antibodies (Hep-2-IgG immunofluorescence); anti-dsDNA (Crithidia luciliae IgG immunofluorescence); anti-Sm IgG-ELISA, Euroimmun, Lübek, Germany); C3 complement (plaque IDR C-3 CAT ND08-12); and C4 complement (plaque IDR C-4 CAT ND08-12), Biocientífica, Curitiba Brazil); anti-Ro/SSA ELISA IgG – ELISA, Euroimmun, Lübek, Germany) and anti-La/SSB (ELISA IgG – ELISA) for primary Sjögren's syndrome, anti-proteinase 3 (Anti-PR3 IgG – ELISA, Euroimmun, Lübek, Germany), anti-myeloperoxidase (Anti-MPO ELISA – IgG – ELISA, Euroimmun, Lübek, Germany) and antineutrophil cytoplasmic antibodies (ANCA) for primary vasculitis and anti-tissue transglutaminase antibodies (IgA, ELISA, Euroimmun, Lübek, Germany) for celiac disease. Hs-CRP was analyzed using Immunochemistry (nephelometry) (Dade Behring; Siemens).

The investigation of these autoantibodies is very important not only as a support for diagnosis but also because they are serological markers that may precede the diagnosis of chronic inflammatory diseases by years, such as anti-Ro/SSA in primary Sjögren's syndrome.^22^, ^23^, ^24^

GlycA, a new inflammatory activity marker, quantifies the acute phase glycoproteins by the magnetic resonance signal of their N-acetyl-methyl group protons on the N-acetylglucosamine (GlcNAc) moieties located on bi-, tri-, or tetra-antennary branches. For lipid tests using Nuclear Magnetic Resonance (NMR), their signal was deconvoluted from overlapping lipoproteins (predominantly triglycerides in very-low-density lipoproteins), and their amplitude converted to micromoles per liter (mmoL/L).^25^ Data were acquired from ethylenediaminetetraacetic acid plasma samples used by LabCorp clinical laboratory in Raleigh, NC. The nuclear magnetic resonance Profiler platform consisted of a 9.4-T (400-MHz 1 Hz frequency) spectrometer with an integrated fluidic sample delivery system. Proprietary deconvolution software was used to quantify the lipid signal.^26^

### Measurement of subclinical vascular disease

Carotid Intima-Media Thickness (CIMT) protocol was published earlier.^27^^,^^28^ The authors defined the average between the mean left and mean right carotid intima-media measurements as CIMT. Briefly, CIMT was measured in the outer wall of a 1 cm predefined carotid segment from 1 cm below the carotid bifurcation during three cardiac cycles. Abnormal CIMT values were defined as those above the 75^th^ percentile. CIMT was also used as a continuous variable. All images were sent to the core reading center in São Paulo and underwent image quality assessment. The authors used the MIA software (Coralville, IA) to standardize the reading and interpretation of valid carotid scans. CIMT was measured at baseline and in visit 3 over a 9-year follow-up allowing prospective analyses. As CIMT was available in subsamples of the main study and after exclusion of participants reporting prevalent CVD at baseline, only 3,246 participants have cIMT information.

Only participants in the research center of São Paulo (n=4547) underwent a Coronary Artery Calcification (CAC) examination performed with a 64-detector computed tomographic scanner (Brilliance 64; Philips Healthcare, Best, The Netherlands). Briefly, each patient underwent an electrocardiogram-gated prospective calcium score examination with a tube potential of 120 kV and a tube current adjusted to body habitus. Images were reconstructed in 2.5 mm slice thickness using standard filtered back projection. The CAC was expressed as Agatston units, and the percentile was evaluated in a blinded fashion by an experienced cardiologist using semiautomatic software (Calcium Scoring, Philips Workstation).^29^ CAC severity was further categorized according to 0 or >0, < 100, or ≥ 100.^30^^,^^31^ CAC images were obtained at baseline and over a 4 year follow-up period. After excluding prevalent cases of CVD at baseline, only 1,325 participants have information about CAC at baseline.

### Lipid paradox

Data from ELSA-Brasil permit the calculation of several risk scores: Framingham risk score, the ASSIGN score,^32^ the QRISK®2 score,^33^ the Systematic Coronary Risk Evaluation (SCORE) algorithm,^34^ the American College of Cardiology and American Heart Association m Pooled cohort Equations (PCE)^35^ and the Reynolds Risk Score (RRS).^36^ The PCE involved a highly admixed sample, and the Reynold Risk Score incorporates the inflammatory marker C-Reactive Protein (CRP) in addition to traditional risk factors. The QRISK®2 score incorporated rheumatoid arthritis as an independent risk factor. This and the possibility to analyze lipid biomarkers using three different techniques (traditional, vertical ultra-centrifugation, and NMR) make it possible to analyze lipid sub-particles and their relationship with the lipid paradox. It is also a great opportunity to study the lipid paradox in other inflammatory diseases beyond rheumatoid arthritis.

### Sociodemographic and clinical variables

Sociodemographic characteristics: sex; age (years); educational attainment (less than high school, high school and some college and at least complete college; mean family monthly income (≤ US$ 1245, US$ 1246 to US$ 3319 and ≥ US$ 3320) and self-reported race/skin color (White, Mixed, Black, Asian and Indigenous). Smoking and alcohol use were categorized as never, past or current.^20^

The anthropometric measures of weight, height, and waist circumference were obtained using international criteria and standards techniques.^37^ The body weight was measured with the subject barefoot, fasted, wearing a standard uniform over his underwear. An electronic scale (Toledo®, model 2096 PP) was used, with a capacity of 200 kg and a precision of 50 g. The height was measured with a wall stadiometer (Seca®, Hamburg, BRD) with a precision of 1 mm, attached to the wall with the individual in the supine position, barefoot, leaning on the head, buttocks, and heels on the wall and with the stare in the horizontal plane. The height was verified in the inspiratory period of the respiratory cycle. Waist Circumference (WC) was measured with the participant in fasting and with the bladder empty, standing upright breathing normally, with the feet together, the part of the dress erected, and with the arms crossed in front of the chest. The measure is made with an inextensible tape measure at the midpoint between the iliac crest and the lower border of the costal arch, and the Body Mass Index (BMI) was calculated as weight (kg) divided by squared height (m^2^).^15^^,^^37^

Blood Pressure (BP) was measured using a validated Omron HEM 705CPINT oscillometric device. Three blood pressure measurements were taken at one-minute intervals, and the mean of the last two measurements was considered as the value for casual systolic or diastolic blood pressure. Hypertension was defined as the use of medication to treat hypertension or systolic blood pressure ≥ 140 mm Hg, or diastolic blood pressure ≥ 90 mm Hg. Diabetes was defined as the previous medical history of diabetes, use of medication to treat diabetes, fasting plasma glucose ≥ 126 mg/dL, 2-hour plasma glucose ≥ 200 mg/dL, or HbA_1C_ ≥ 6.5%. Dyslipidemia was defined as LDL-cholesterol ≥ 130 mg/dL or use of any lipid-lowering medication. Thyroid function was defined based on TSH, FT4, FT3 levels, and the use of levothyroxine or thiamazole. Leisure-time physical activity was classified according to the World Health Organization criterion using the International Physical Activity Questionnaire (IPAQ), in which physically active were those with at least 150 minutes of moderate-intensity or 75 minutes of high-intensity leisure-time aerobic physical activity or the combined equivalent of both each week. Any weekly activity below the described threshold was classified as partly active, and the remaining participants were classified as sedentary.^38^ For mental health it was used the Clinical Interview Schedule Questionnaire, version Revised (CIS-R) elaborated by Lewis et al., in 1992 for diagnosis of non-psychotic psychiatric diagnosis at primary care.^39^ The answer to the questionnaire permits psychiatric classification diagnosis according to the International Classification of Diseases version 10^40^ as common mental health, depressive disorder, and generalized anxiety disorders.

### Other laboratory tests

#### Thyroid function

Venous blood samples were drawn after an overnight fast and centrifuged at 2500g for 15 min to obtain serum for biochemistry and determination of hormone levels.

The study includes information about fasting plasma glucose (Hexokinase method; ADVIA Chemistry; Siemens, Deerfield, Illinois), 2h plasma glucose after a glucose overload (Oral Glucose Tolerance Test ‒ OGTT), glycated hemoglobin (High pressure liquid chromatography, Bio-Rad Laboratories, Hercules, California), fasting insulin and 2h insulin (Immunoenzymatic assay, ELISA, Siemens), total cholesterol, and HDL cholesterol (Enzymatic colorimetric assay, ADVIA Chemistry), LDL cholesterol (Calculated by means of the Friedewald equation), triglycerides (Enzymatic colorimetric assay, glycerol phosphate peroxidase, ADVIA Chemistry), high sensitivity C-Reactive protein (Immunochemistry nephelometry), creatinine (Enzymatic colorimetric assay, Jaffé, ADVIA Chemistry), liver enzymes (Alanine Aminotransferase ‒ ALT, Aspartate Aminotransferase ‒ AST, (Modified International Federation for Clinical Chemistry [enzymatic] assay, ADVIA Chemistry) and Gamma-Glutamil Transpeptidase ‒ GGT (Kinetic colorimetric assay), uric acid (Enzymatic colorimetric assay), ADVIA Chemistry, thyroid function (Thyroid-Stimulating Horomone ‒ TSH, Free Thyroxine ‒ FT4 and Free Triiodothyronine ‒ FT3) and Thyroperoxidase Antibodies ‒ TPOAb, (for all of them, Roche Diagnostics, Manheim, Germany) total blood cell count, microabuminuria Immunochemistry (nephelometry) (Dade Behring; Siemens), urinary sodium and potassium (Potentiometry (ion-selective electrodes) (ADVIA Chemistry), and serology for Chagas disease. (Microplate ELISA (Chagatest ELISA; Wiener Laboratories, Rosario, Argentina).^41^ Beyond these classical tests, ELSA-Brasil also has information about lipids measured with innovative techniques such as Nuclear Magnetic Resonance (NMR) and vertical ultracentrifugation.

#### Stored samples

ELSA-Brasil has a central biorepository in the Research Center of São Paulo. Since 2008 serum, plasma, and urine samples of all participants have been stored in nitrogen to be used in future analysis.^42^

### Statistical analysis

#### Descriptive characteristics at baseline

Categorical variables are presented as proportions and compared using the chi-squared test. Continuous variables, if normally distributed, are presented as mean (standard deviation) and compared using ANOVA; if non-normal, they will be presented as median (interquartile range) and compared using Mann-Whitney or Kruskall-Wallis tests as indicated.

Future analyses related to the associations between CID and CVD:

Cross-sectional associations of CID with CVD will be analyzed using logistic regression models. The results will be presented as Odds Ratio (OR) and respective 95% Confidence Intervals (95% CI) both as crude (non-adjusted) and adjusted for sociodemographic variables (age, sex, self-reported race, education, and mean monthly family net income). Further multivariate adjustment for cardiovascular disease risk factors (hypertension, diabetes, dyslipidemia, smoking, alcohol use, and physical activity) and specific confounders determined for each analysis will be performed.

For prospective associations, the authors will use weighted Cox-proportional hazards according to the Barlow method presented as Relative Risk (RR) with respective 95% CI crude, adjusted for sociodemographic models, and with multivariate adjustment for confounders as described above.^43^ In some cases, Poisson regression with robust variance to determine the Relative Risk (RR) with respective 95% CI, both as crude estimates and with the multivariate adjustment presented above. The association of inflammatory diseases with fatal and non-fatal cardiovascular events and mortality will be analyzed using Kaplan-Meier curves (compared using the log-rank test).

## Results

**Figure 1:**
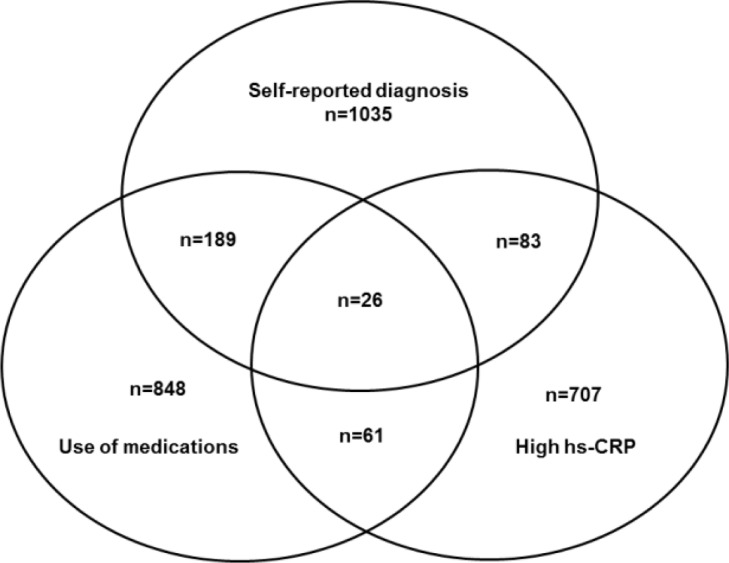
Venn diagram showing the selection of participants to the high-risk groups for chronic inflammatory diseases according to the number of criteria.

Figure 1 describes in a Venn diagram the selection of participants in the high-risk group according to previous self-reported medical diagnosis, and/or use of medication to treat CID and/or hsCRP > 10 mg/L in one or more than one Visit: 26 participants present all 3 criteria, 333 presented 2 criteria, and 2589 presented just one criterion (Fig. 1)

Table 1 shows sociodemographic and clinical characteristics of the sample according to the high risk of CID and the ACS. Participants in the group with high-risk of CID were older (p < 0.0001), mostly women (p < 0.0001) with a higher frequency of Blacks and a lower frequency of Asians (p = 0.04), presented low education attainment (p = 0.005), lower average net monthly income (p < 0.0001), a higher frequency of never or past alcohol intake (p = 0.005), a higher BMI and waist circumference (for both p < 0.0001), and a higher frequency of hypertension (p < 0.0001), diabetes (p = 0.003) and depression (p = 0.007). Levels of GlycA (p < 0.0001) were higher compared to participants in the ACS.

**Table 1:** Sociodemographic and clinical characteristics from the participants of the ELSA-Brasil study according to the risk of inflammatory diseases (high-risk cases and aleatory cohort sample).

	Aleatory cohort sample (n = 1,543)	Inflammatory diseases (n = 2,949)	p
**Age (years)**	52.2 ± 8.8	53.6 (9.2)	< 0.0001
**Women (%)**	834 (54.1)	1932 (65.5)	< 0.0001
**Race (%)**			0.04
White	772 (50.4)	1454 (49.8)	
Mixed	418 (27.3)	824 (28.2)	
Black	271 (17.7)	558 (19.1)	
Asian	50 (3.3)	55 (1.9)	
Indigenous	20 (1.3)	30 (1)	
**Education (%)**			0.005
Less than high school	175 (11.3)	412 (14)	
High school and some College	523 (33.9)	1058 (35.8)	
Complete College or more	845 (54.8)	1479 (50.2)	
Average family monthly income (%)			< 0.0001
<US$ 1245	382 (24.8)	856 (29.1)	
US$ 1245 – 3320	561 (36.5)	1139 (38.7)	
>US$ 3320	585 (38.7)	946 (32.2)	
**Smoking (%)**			0.47
Never	866 (56.2)	1600 (54.3)	
Past	490 (31.7)	982 (33.3)	
Current	186 (12.1)	367 (12.4)	
**Alcohol intake**			0.005
Never	146 (9.4)	341 (11.5)	
Past	314 (20.4)	675 (23)	
Current	1082 (70.2)	1925 (65.5)	
**Body-mass Index (kg/m^2^)**	26.9 ± 4.6	28.1 ± 5.5	< 0.0001
**Waist circumference (cm)**	91 ± 12.8	93.3 (13.7)	< 0.0001
**Mean systolic blood pressure (mm Hg)**	121 ± 16	122 ± 18	0.09
**Mean diastolic blood pressure (mm Hg)**	76 ± 10	76.4 ± 11	0.32
**Hypertension (%)**	532 (34.5)	1184 (40.2)	< 0.0001
**Diabetes (%)**	263 (17)	610 (20.7)	0.003
**Dyslipidemia %)**	705 (45.8)	1386 (47.2)	0.37
**Depression (%)**	60 (3.9)	170 (5.8)	0.007
**Thyroid function**			0.053
Overt hyperthyroidism	5 (0.3)	26 (0.9)	
Subclinical hyperthyroidism	26 (1.7)	38 (1.3)	
Euthyroidism	1301 (84.5)	2431 (82.6)	
Subclinical hypothyroidism	86 (5.6)	164 (5.6)	
Overt hypothyroidism	121 (7.9)	281 (9.6)	
**Fasting plasma glucose**	106 ± 27	108 ± 30	0.09
**2h plasma glucose mg/dL**	204 ± 83	222 ± 95	0.11
**Total Cholesterol mg/dL**	200 ± 41	202 ± 44	0.12
**HDL - Cholesterol mg/dL**	53.8 ± 13.2	54.3 ± 13.6	0.19
**LDL - Cholesterol mg/dL**	118.8 ± 35.4	119.4 ± 34.9	0.57
**Triglycerides mg/dL**	126 ± 74	130 ± 105	0.42
**High sensitivity C Reactive protein mg/L**	2,56 ± 3,29	5,65 ± 8.64	< 0.0001
**Glyc A µmoL/L**	413 ± 65	443 ± 77	< 0.0001
**Mean carotid IMT mm**	0.60 ± 0.13	0.62 ± 0.13	< 0.0001
**CAC >0 (%) Agatston units**	156 (30.3)	221 (17.3)	0.24

IMT, Intima-Media of carotid; CAC, Coronary Artery Calcium.

Two surrogate markers for subclinical atherosclerosis were compared between both groups after excluding participants reporting prevalent cardiovascular diseases at baseline (myocardial infarction, revascularization, and stroke). After exclusions, cIMT was available in 3,246 participants. Mean Carotid IMT (CIMT) was higher in participants in the CID group compared to the ACS (p < 0.0001). Regarding CAC, after exclusions, 1,325 participants had CAC measurements available at baseline. There was no difference regarding the percentage of CAC > 0 between groups.

## Discussion

Comparisons between the CID and the ACS groups suggest that the study's proposed strategy to identify CID was effective. Participants in the high-risk group for the inflammatory disease were older, mostly women, with low education attainment and low socioeconomic status. Anthropometric markers such as BMI and waist circumference were higher in the CID group as well as the levels of GlycA. Beyond that, participants in the high-risk group presented a higher prevalence of hypertension, diabetes, depression, and a trend to thyroid diseases. For subclinical atherosclerosis biomarkers, mean CIMT was higher in the high-risk group, but no difference was detected for CAC. As expected, some participants were selected by more than one criterion. These data suggest that the strategy used to identify the high-risk group was effective. Both groups will be adequate for further testing of specific biomarkers for each inflammatory disease in this subsample.

The study has some characteristics that need to be highlighted. Two epidemiologic designs are possible in cohort studies that have stored biological samples: the nested case-control study and the case-cohort study. The most used is the nested case-control study. In the follow-up of a cohort study, several diseases are going to be diagnosed. Researchers can assign one or more controls selected according to sociodemographic and clinical characteristics similar to the cases for each analysis to be done. Biomarkers related to the disease can be tested in all cases, and a selected smaller group of comparison paired by age and sex would be tested in the place of the entire cohort. Therefore, the costs of tests become less expensive than including the entire cohort. An example of success using this strategy is an article focused on the association of inflammatory biomarkers in women selected after a cardiovascular event and a comparison paired group in the Women's Health Initiative (WHI). The inflammatory biomarkers were tested only in 304 women that developed cardiovascular events at follow-up and 304 women without any cardiovascular events chosen as the comparison group. Considering that the study had 75,343 participants, this approach was more cost-effective than testing the entire cohort. The results showed that women with higher levels of hs-CRP at baseline present a higher risk of a cardiovascular event at follow-up.^44^ One limitation of this type of design is that it is necessary to define a new group of comparison paired at least by age and sex for each performed analysis. Compared to the nested case-control design, the case-cohort design was proposed to minimize this problem.^45^ At the beginning of the cohort study, a representative subsample of participants can be randomly selected.^21^ This subsample of generally 10% to 15% of the entire sample has more biological samples collected and stored at any phase of the study. Therefore, in a case-cohort study, this subsample of participants can be the comparison group for different analysis. This strategy reduces costs, maintaining the cohort representativeness. In the present analysis, only 2,949 participants in the CID group and 1,543 of the ACS, totaling 4,492 participants, will be tested as opposed to all 15,105 cohort participants. Thus, the authors chose to use a case-cohort design because of the many advantages offered by this design in terms of less expensive costs, the simplified logistics of using the same comparison group for all analyses as well as the possibility to study many diseases with the same comparison group.

The present study's strategy in the analysis for each type of inflammatory disease is similar to a previous analysis used to identify rheumatoid arthritis in the WHI. In this study, women who self-report a previous medical diagnosis of rheumatoid arthritis or that use Disease-Modifying Anti-Rheumatic Drugs (DMARDs) were selected as cases to be tested for anti-CCP antibodies and Rheumatoid Factor. In the end, 11,017 women were tested of the 161,808 participants of WHI. The authors reported an almost two times higher mortality in women with rheumatoid arthritis compared to controls.^8^ They used as comparison group women reporting arthritis but not rheumatoid arthritis (n = 57,572) and a second group of women reporting no arthritis (n = 76,160). Participants in both comparison groups were not tested for any biomarker. After the results of biomarkers, the final diagnosis of probable rheumatoid arthritis was made considering all women with anti-CCP positive and women with anti-CCP negative but using DMARDS; women that reported rheumatoid arthritis but presented negative anti-CCP and were not using DMARDS were classified in the group of not likely clinical rheumatoid arthritis. Compared to the WHI study, in the present analyses, the authors will use a case-cohort strategy that included biomarker testing in the CID and the comparison group that will be the same for all diseases included as part of the project. The authors have information about women reporting arthritis not related to rheumatoid arthritis. Therefore, the authors can use the same strategy used in the WHI of using one group of comparison reporting arthritis not related to rheumatoid arthritis and one group reporting no arthritis. The authors have the possibility to use different strategies in the analysis of CID, which brings versatility to the study and the possibility to explore the same data in different ways. For diseases that did not present specific biomarkers as is the case of psoriasis, in which the gold-standard for diagnosis is a prior medical diagnosis, it is possible to include all the other participants in the study without CID as the comparison group or using new techniques of paired analysis as the propensity score matching.

This protocol is an excellent opportunity to study the lipid paradox using new techniques to measure lipids such as by RNM or ultra-vertical centrifugation that are not frequently available in cohort studies. As several diseases will be studied simultaneously, one of the advantages of the study design is the possibility of spreading information about the lipid paradox originally described for rheumatoid arthritis to other inflammatory diseases.

The strength of the analysis is to use data from the ELSA-Brasil study with information collected under strict quality control protocols after centralized training of all study teams before the periods of acquisition of data. Most cohort studies on CID were performed in high-income countries, and there is scarce information about inflammatory diseases in LMIC. ELSA-Brasil is an excellent opportunity to evaluate CID and its relationship with cardiovascular disease, cardiovascular events, and all-cause, cardiovascular, and other specific causes of mortality. For example, it is possible to use this data to study gastro-intestinal mortality associated with celiac disease in a multiethnic sample from an LMIC. The study also has some limitations related to the possible underestimation of cases of mild CID that may not be selected in the high-risk group and will be out of the analysis, overestimating the association of CID with cardiovascular disease, events, and mortality that tend to be lower in mild cases. In addition, even in a large sample, as the prevalence of some diseases included in the analysis is low, it is possible that the authors were not able to conduct some specific analysis for diseases with s small number of cases, as is the case of primary vasculitis

In conclusion, the strategy to select the sample and the design to be used in the analysis is a very cost-effective strategy, and this is a significant approach in a country with limited funding for research compared to high-income countries. Moreover, data from this project will bring new information about the cross-sectional and prospective association of CID and the increased risk of CVD events and mortality, the most common cause of death, and a strong burden of related disabilities in the country.

## Authors' contributions

IMB (conception, design, analysis, interpretation of data, draft and final version, funding) ACG (interpretation of data, critical review, draft, final version), ACP (interpretation of data, draft, critical review), ARB, AA, RDS, MSB, RWT, LACM, SMB, BAP, CPSJ, JAS, WRT, VM, FBJ, ACMR, SGP, RMRP, EB e AMS (interpretation of data, critical review), ISS (interpretation of data, critical review, draft, final version), PAL (interpretation of data, critical review, funding). All authors have reviewed and approved the final version of the manuscript.

## Conflicts of interest

The authors declare no conflicts of interest.

## References

[1] Roth GA, Mensah GA, CO Johnson, Addolorato G, Ammirati E, Baddour LM (2020). Global Burden of Cardiovascular Diseases and Risk Factors, 1990-2019: Update From the GBD 2019 Study. J Am Coll Cardiol.

[2] GBD 2017 Causes of Death Collaborators (2018). Global, regional, and national age-sex-specific mortality for 282 causes of death in 195 countries and territories, 1980-2017: a systematic analysis for the Global Burden of Disease Study 2017. Lancet.

[3] Brant LCC, Nascimento BR, Passos VMA, Duncan BB, Bensenor IM, Malta DC (2017). Variations and particularities in cardiovascular disease mortality in Brazil and Brazilian states in 1990 and 2015: estimates from the Global Burden of Disease. Rev Bras Epidemiol.

[4] Lotufo P. (2015). Cardiovascular disease in Brazil: premature mortality, risk factors and priorities for action. Comments on the preliminary results from the Brazilian National Health Survey (PNS), 2013. Sao Paulo Med J.

[5] Abdul Sultan A, Crooks CJ, Card T, Tata LJ, Fleming KM, West J (2015). Causes of death in people with coeliac disease in England compared with the general population: a competing risk analysis. Gut.

[6] Libby P, Loscalzo J, Ridker PM, Farkouh ME, Hsue PY, Fuster V (2018). Inflammation, immunity, and infection in atherothrombosis: JACC review topic of the week. J Am Coll Cardiol.

[7] Ridker PM, Hennekens CH, Buring JE (2000). Rifai N. C-reactive protein and other markers of inflammation in the prediction of cardiovascular disease in women. N Engl J Med.

[8] Kuller LH, Mackey RH, Walitt BT, Deane KD, Holers VM, Robinson WH (2014). Determinants of mortality among postmenopausal women in the 'women's health initiative who report rheumatoid arthritis. Arthritis Rheumatol.

[9] Gu MM, Wang XP, Cheng QY, Zhao YL, Zhang TP, Li BZ (2019). A meta-analysis of cardiovascular events in systemic lupus erythematosus. Immunol Invest.

[10] Cooksey R, Brophy S, Kennedy J, Gutierrez FF, Pickles T, Davies R (2018). Cardiovascular risk factors predicting cardiac events are different in patients with rheumatoid arthritis, psoriatic arthritis, and psoriasis. Semin Arthritis Rheum.

[11] Boo S, Oh H, Froelicher ES, Suh CH. (2017). Knowledge and perception of cardiovascular disease risk among patients with rheumatoid arthritis. PLoS One.

[12] Behl T, Kaur I, Sehgal A, Zengin G, Brisc C, Brisc MC (2020). The lipid paradox as a metabolic checkpoint and its therapeutic significance in ameliorating the associated cardiovascular risks in rheumatoid arthritis patients. Int J Mol Sci.

[13] Giles JT, Wasko MCM, Chung CP, Szklo M, Blumenthal RS, Kao A (2019). exploring the lipid paradox theory in rheumatoid arthritis: associations of low circulating low-density lipoprotein concentration with subclinical coronary atherosclerosis. Arthritis Rheumatol.

[14] Myasoedova E, Crowson CS, Kremers HM, Roger VL, Fitz-Gibbon PD, Therneau TM (2011). Lipid paradox in rheumatoid arthritis: the impact of serum lipid measures and systemic inflammation on the risk of cardiovascular disease. Ann Rheum Dis.

[15] Aquino EM, Barreto SM, Benseñor IM, Carvalho MS, Chor D, Duncan BB (2012). Brazilian Longitudinal Study of Adult Health (ELSA-Brasil): objectives and design. Am J Epidemiol.

[16] Laurent S, Alivon M, Beausiier H, Boutouyrie P. (2012). Aortic stiffness as a tissue biomarker for predicting future cardiovascular events in asymptomatic hypertensive subjects. Ann Med.

[17] Cai R, Wu X, Li C, Chao J. (2020). Prediction models for cardiovascular disease risk in the hypertensive population: a systematic review. J Hypertens.

[18] Schmidt MI, Duncan BB, Mill JG, Lotufo PA, Chor D, Barreto SM (2015). Cohort Profile: Longitudinal Study of Adult Health (ELSA-Brasil). Int J Epidemiol.

[19] Bensenor IM, Griep RH, Pinto KA, Faria CP, Felisbino-Mendes M, Caetano EI (2013). Routines of organization of clinical tests and interviews in the ELSA-Brasil investigation center. Rev Saude Publica.

[20] Chor D, Alves MG, Giatti L, Cade NV, Nunes MA, Molina Mdel C (2013). Questionnaire development in ELSA-Brasil: challenges of a multidimensional instrument. Rev Saude Publica.

[21] Onland-Moret NC, van der A DL, van der Schouw YT, Buschers W, Elias SG, van Gils CH (2007). Analysis of case-cohort data: a comparison of different methods. J Clin Epidemiol.

[22] Rivera TL, Izmirly PM, Birnbaum BK, Byrne P, Brauth JB, Katholi M (2009). Disease progression in mothers of children enrolled in the Research Registry for Neonatal Lupus. Ann Rheum Dis.

[23] Jonsson R, Theander E, Sjöström B, Brokstad K, Henriksson G. (2013). Autoantibodies present before symptom onset in primary Sjögren syndrome. JAMA.

[24] Theander E, Jonsson R, Sjöström B, Brokstad K, Olsson P, Henriksson G. (2015). Prediction of Sjögren's syndrome years before diagnosis and identification of patients with early onset and severe disease course by autoantibody profiling. Arthritis Rheumatol.

[25] Fournet B, Montreuil J, Strecker G, Dorland L, Haverkamp J, Vliegenthart FG (1978). Determination of the primary structures of 16 asialo-carbohydrate units derived from human plasma alpha 1-acid glycoprotein by 360-MHZ 1H NMR spectroscopy and permethylation analysis. Biochemistry.

[26] Jeyarajah EJ, Cromwell WC, Otvos JD. (2006). Lipoprotein particle analysis by nuclear magnetic resonance spectroscopy. Clin Lab Med.

[27] Mill JG, Pinto K, Griep RH, Goulart A, Foppa M, Lotufo PA (2013). Medical assessments and measurements in ELSA-Brasil. Rev Saude Publica.

[28] Santos IS, Bittencourt MS, Oliveira IR, Souza AG, Meireles DP, Rundek T (2014). Carotid intima-media thickness value distributions in the Brazilian Longitudinal Study of Adult Health (ELSA-Brasil). Atherosclerosis.

[29] Agatston AS, Janowitz WR, Hildner FJ, Zusmer NR, Viamonte M, Detrano R. (1990). Quantification of coronary artery calcium using ultrafast computed tomography. J Am Coll Cardiol.

[30] Pereira AC, Gomez LM, Bittencourt MS, Staniak HL, Sharovsky R, Foppa M (2016). Age, gender, and race-based coronary artery calcium score percentiles in the Brazilian Longitudinal Study of Adult Health (ELSA-Brasil). Clin Cardiol.

[31] Bensenor IM, Goulart AC, Santos IS, Bittencourt MS, Pereira AC, Santos RD (2016). Association between a healthy cardiovascular risk factor profile and coronary artery calcium score: results from the Brazilian Longitudinal Study of Adult Health (ELSA-Brasil). Am Heart J.

[32] Tunstall-Pedoe (2005).

[33] Hippisley-Cox J, Coupland C, Vinogradova Y, Robson J, May M, Brindle P. (2007). Derivation, and validation of QRISK, a new cardiovascular disease risk score for the United Kingdom: prospective open cohort study. BMJ.

[34] Conroy RM, Pyorala K, Fitzgerald AP, Sans S, Menotti A, De Backer G (2003). Estimation of ten-year risk of fatal cardiovascular disease in Europe: the SCORE project. Eur Heart J.

[35] Goff DC, Lloyd-Jones DM, Bennett G, Coady S, 'D'Agostino RB Sr, Gibbons R (2014). 2013 ACC/AHA guideline on the assessment of cardiovascular risk: a report of the American College of Cardiology/American Heart Association Task Force on Practice Guidelines. J Am Coll Cardiol.

[36] Ridker PM, Buring JE, Rifai N, Cook NR. (2007). Development and validation of improved algorithms for the assessment of global cardiovascular risk in women: the Reynolds Risk Score. JAMA.

[37] Lohman TG, Roche AF, Martorell R. Anthropometric standardization reference manual. Human Kinetics Books, Champaign, IL1988.

[38] Treff C, Bensenor IM, Lotufo PA. (2017). Leisure-time and commuting physical activity and high blood pressure: the Brazilian Longitudinal Study of Adult Health (ELSA-Brasil). J Hum Hypertens.

[39] Lewis G, Pelosi AJ, Araya R, Dunn G. (1992). Measuring psychiatric disorder in the community: a standardized assessment for use by lay interviewers. Psychol Med.

[40] World Health Organization. ICD-10: international statistical classification of diseases and related health problems: tenth revision, 2nd ed. 2004. https://apps.who.int/iris/handle/10665/42980.

[41] Fedeli LG, Vidigal PG, Leite CM, Castilhos CD, Pimentel RA, Maniero VC (2013). Logistics of collection and transportation of biological samples and the organization of the central laboratory in the ELSA-Brasil. Rev Saude Publica.

[42] Pereira AC, Bensenor IM, Fedeli LM, Castilhos C, Vidigal PG, Maniero V (2013). Design and implementation of the ELSA-Brasil biobank: a prospective study in a Brazilian population. Rev Saude Publica.

[43] Barlow WE, Ichikawa L, Rosner D, Izumi S. (1999). Analysis of case-cohort designs. J Clin Epidemiol.

[44] Pradham AD, Manson JE, Rossouw JE, Siscovick DS, Mouton CP, Rifai N (2002). Inflammatory biomarkers, hormone replacement therapy, and incident coronary heart disease: prospective analysis from the Women's Health Initiative observational study. JAMA.

[45] Prentice RL. (1986). A case-cohort design for epidemiologic cohort studies and disease prevention trials. Biometrika.

